# Results of Hepatectomy for Hepatocellular Carcinoma at the National Cancer Center Hospital

**DOI:** 10.1155/1991/54751

**Published:** 1991

**Authors:** Susumu Yamasaki, Masatoshi Makuuchi, Hiroshi Hasegawa

**Affiliations:** 1 Department of Surgical Oncology National Cancer Center Hospital Tokyo Japan; 2 Department of Surgical Oncology National Cancer Center Hospital 5-1-1 Tsukiji Chuo-ku Tokyo 104 Japan

## Abstract

The number of hepatectomies has increased greatly in recent years. Surgery for hepatocellular carcinoma (HCC) in the normal liver has not increased. However, the increase in numbers of hepatectomies for HCC associated with liver cirrhosis is remarkable. More than 80% of our hepatectomy cases were cirrhotic and about 80% of these cirrhotic cases had HCCs 5cm or less in diameter. The operative mortality rate has improved in the latter half of this series, from 10.1% (9/89) to 1.5% (5/338), in spite of an increase in cases with poor liver function. This corresponds to a decrease in the mean value of the annual operative blood loss. The survival rates after hepatectomy for all cases (n = 378) were 40.6% ± 6.6 (% ± SE) for 5 year and 22.7% ± 5.3 for 10 year at the end of 1988. A difference of the 5-year survival rate between the patients operated on before 1981 (n = 78, 25.6% ± 4.9) and after 1982 (n = 300, 46.1% ± 4.8) was observed (p<0.05). Because the cancer-free survival rates of the patients operated on in the two periods, before 1981 and after 1982, were almost the same, the recent improvement of the survival rates seems to be due to a prolongation of survival time after recurrence.


*HPB Surgery*, 1991, Vol. 3, pp. 235–249
Reprints available directly from the publisher
Photocopying permitted by license only

© 1991 Harwood Academic Publishers GmbH
Printed in the United Kingdom

### RESULTS OF HEPATECTOMY FOR HEPATOCELLULAR CARCINOMA AT THE NATIONAL CANCER CENTER HOSPITAL

SUSUMU YAMASAKI, MASATOSHI MAKUUCHI and HIROSHI HASEGAWA
*Department of Surgical Oncology, National Cancer Center Hospital, Tokyo*

*(Received 1 July 1990)*

The number of hepatectomies has increased greatly in recent years. Surgery for hepatocellular carcinoma (HCC) in the normal liver has not increased. However, the increase in numbers of hepatectomies for HCC associated with liver cirrhosis is remarkable. More than 80% of our hepatectomy cases were cirrhotic and about 80% of these cirrhotic cases had HCCs 5cm or less in diameter. The operative mortality rate has improved in the latter half of this series, from 10.1% (9/89) to 1.5% (5/338), in spite of an increase in cases with poor liver function. This corresponds to a decrease in the mean value of the annual operative blood loss. The survival rates after hepatectomy for all cases (n = 378) were 40.6% ± 6.6 (% ± SE) for 5 year and 22.7% ± 5.3 for 10 year at the end of 1988. A difference of the 5-year survival rate between the patients operated on before 1981 (n = 78, 25.6% ± 4.9) and after 1982 (n = 300, 46.1% ± 4.8) was observed ($p < 0.05$). Because the cancer-free survival rates of the patients operated on in the two periods, before 1981 and after 1982, were almost the same, the recent improvement of the survival rates seems to be due to a prolongation of survival time after recurrence.

KEY WORDS: Hepatocellular carcinoma, hepatectomy, operative mortality rate, survival rate, cancer-free survival

### INTRODUCTION

From the middle of the 1970s, hepatectomy for hepatocellular carcinoma (HCC) has been the routine clinical practice. Although the numbers of hepatectomies during the 1970s were not many, recently the numbers have increased remarkably. Modern technology has provide some weapons in the field of medicine such as ultrasonography(US) computerized tomography (CT) and some new surgical instruments. These new techniques have allowed the early detection of HCC. Many operable cases of HCC were detected during a follow-up survey for chronic liver diseases. The effects of these changes seems to occur after 1981. This paper reports the results of hepatectomy for HCC with special reference to the annual trends in hepatic oncology.

Correspondence to: Susumu Yamasaki, Department of Surgical Oncology, National Cancer Center Hospital, 5-1-1 Tsukiji, Chuo-ku, Tokyo, JAPAN 104

### PATIENTS AND METHODS

The subject of this report is 427 patients with HCC treated by hepatectomy between October, 1974 and December, 1988 at the National Cancer Center Hospital. In the analysis of survival, cases of non-curative resection or re-resection are excluded. A non-curative resection is defined here as a resection in which some macroscopic tumor or tumor demonstrated by imaging is not removed at laparotomy. Eleven cases of the mixed-type of hepatocellular and cholangiocellular liver cancer are included in this series.

### RESULTS

### *Number of Hepatectomies*

Until the end of 1988, a total of 664 hepatectomies were completed including 427 HCC and 237 liver tumors of other kinds as shown in Table 1. HCC with liver cirrhosis (LC) constituted more than half of the cases, and the second largest group was liver metastases including about 30 cases from a non-splanchnic origin. HCC without LC was only 11% (73/664) of the cases. There were some other kinds of liver tumors in this series, such as children's liver tumors (23), benign liver tumors (36), Klatskin tumors (26), cholangiocellular carcinomas (8), gall bladder cancers (11) and one other rare liver maalignancy (sclerosing epithelioid hemangiosarcoma). The annual number of hepatectomies at our hospital, for all hepatic diseases is shown in Figure 1. Cases are divided into three categories, HCC in the normal liver [HCC,LC(−)], HCC with liver cirrhosis [HCC, LC(+)] and other liver tumors. "Cirrhosis" here means "injured liver" which includes not only true cirrhosis, but also precirrhosis and chronic active hepatitis.

### *Size and number of HCC nodules*

Trends in the size of HCC nodules treated by hepatectomy each year are demonstrated in Figure 2. The greatest dimension of the biggest tumor in each case

**Table 1** Number of hepatectomy for various kinds of hepatic disease

| *Liver Tumor* | *No. of Cases (%)*[b] |
|---|---|
| Hepatocellular carcinoma, with cirrhosis | 354 (53.3%) |
| Hepatocellular carcinoma, without cirrhosis | 73 (11.0%) |
| Liver metastases | 132 (19.9%) |
| Benign tumor | 36 (5.4%) |
| Klatskin tumor | 26 (3.9%) |
| Children's liver malignancy | 23 (3.5%) |
| Gall bladder cancer | 11 (1.7%) |
| Cholangiocellular carcinoma | 8 (1.2%) |
| other [a] | 1 (0.2%) |
| Total | 664 (100%) |

[a] Sclerosing epithelioid hemangiosarcoma
[b] Cases operated on before the end of 1988.

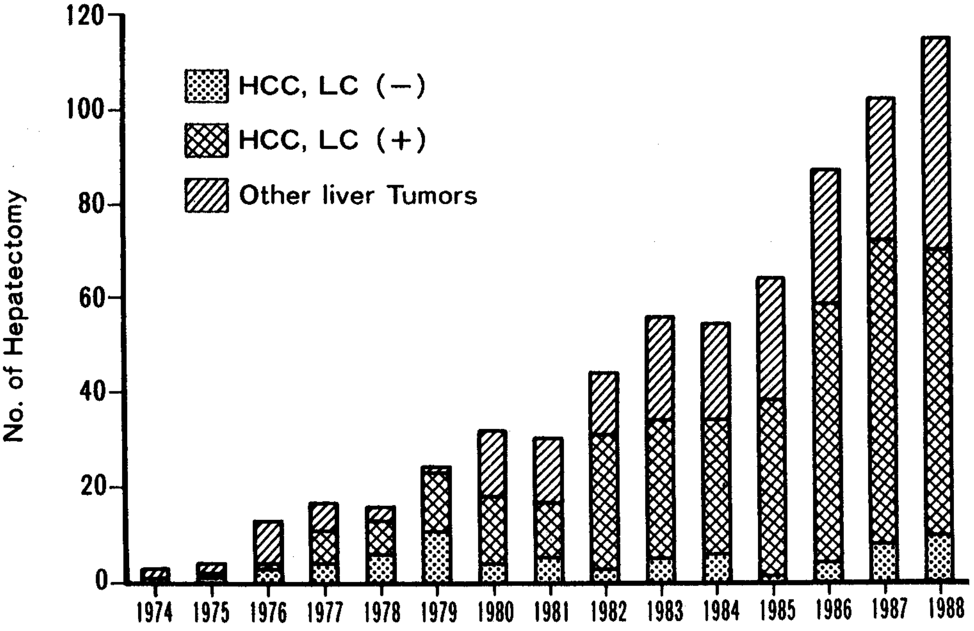

**Figure 1** Annual trends in the number of hepatectomies carried out at the National Cancer Center hospital. HCC, LC(−) means Hepatocellular carcinoma in the normal liver and HCC, LC( + ) means that associated with liver cirrhosis, precirrhosis or chronic active hepatitis. Other tumors includes other kinds of malignancies or benign tumors listed in Table 1.

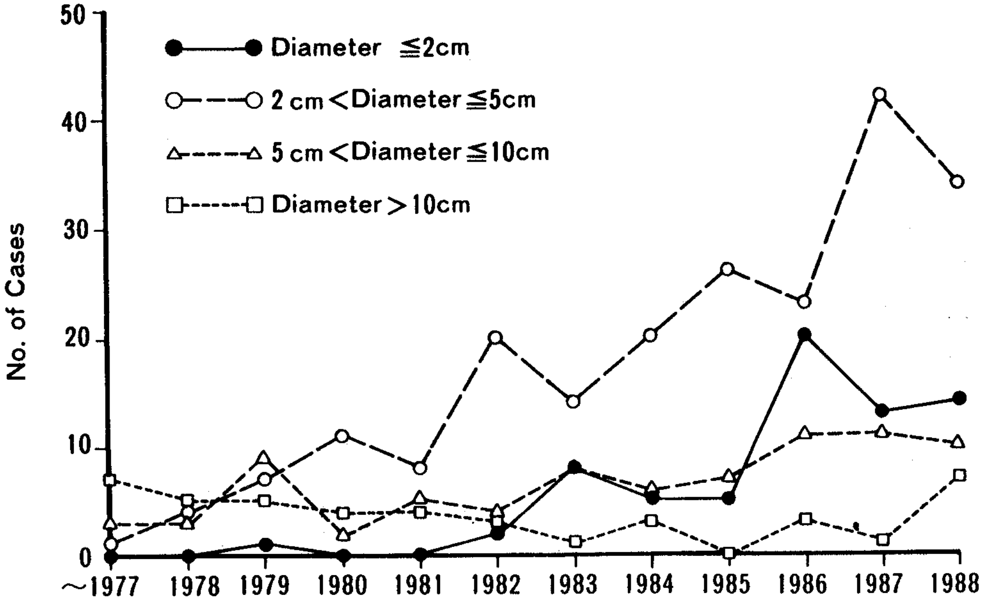

**Figure 2** The numbers of hepatectomized cases in each year are shown divided according to the largest diameter of the largest deposit of HCC(s) in each case.

represents the "size" of the case. The sizes of the groups are as follow: (1)2cm or less, (2)>2cm and ≤5cm, (3)>5cm and ≤10cm and (4)more than 10cm. The numbers in the larger size groups, (3) and (4), are almost stable for the whole period of this series. However, those of the smaller, (1) and (2), have been increasing year by year recently. The proportions of the number of HCC nodule(s), solitary or multiple, in these four size groups and in overall cases were almost the same. Thus, the solitary cases were 215 and the multiples were 212, as shown in Figure 3.

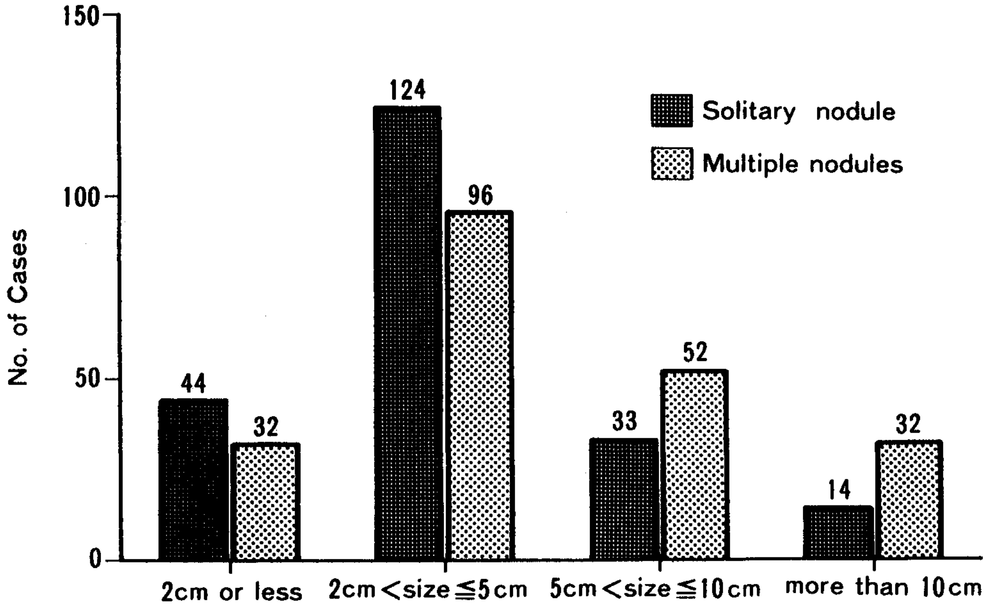

**Figure 3** The numbers of cases with HCC(s) in each size group by solitary or multiple nodules. The total number of cases with solitary nodule was 215/427 (50.4%) and that of multiple nodules was 212/427 (49.6%).

### *Liver function*

There have been several classifications of liver function, such as that of Child[1]. But these classifications of liver function involve all patients with liver diseases from those with almost normal liver function to those in the end stage of the disease. Generally, the patients in whom hepatectomy is indicated have rather good liver function. Child's A or at worst the better part of Child's B. Surgeons, want a classification which categorize patients with good liver function to help in deciding on indications for operation and selection of surgical procedure. Patients, are divided into three functional groups, A, B, and C by the value of total serum bilirubin (T. Bil.) and that of Indocyaningreen (ICG) 15 minutes retention rate [ICG(15′)], "Function A" is with 1.0mg/dl or less for T. Bil. and 15% or less for ICG(15′). "Function B" is with "T. Bil. >1 and ≤2.0mg/dl and ICG(15′)≤40%" and "function C" is with <T. Bil. >2mg/dl or ICG(15′) >40% as shown in Figure 4.

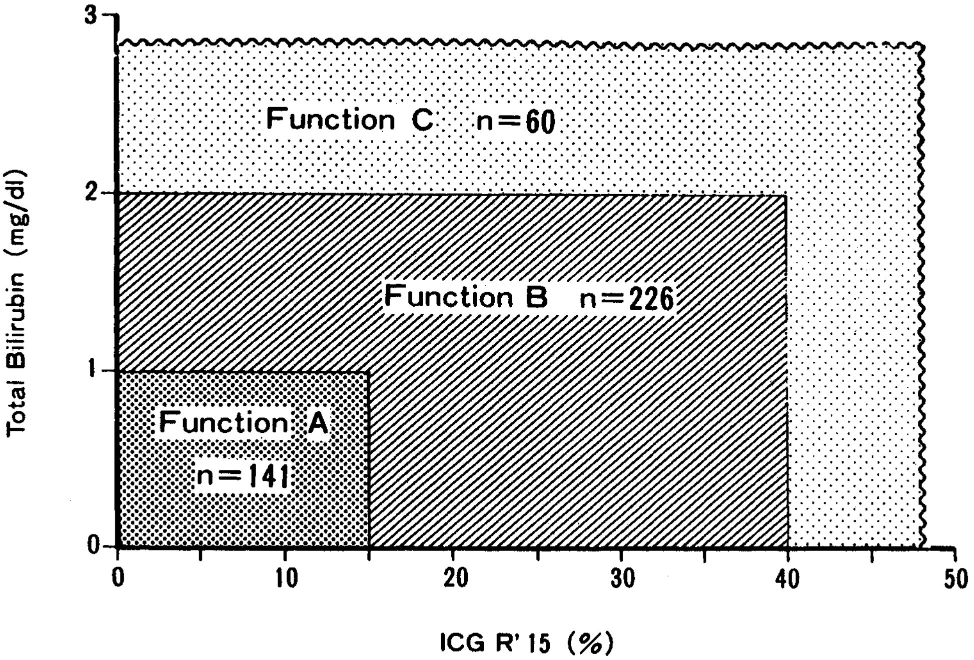

**Figure 4** A classification of liver function by total serum bilirubin and Indocyanin-green (ICG) 15 minutes retention rate. "Function A": almost normal function, "Function B": moderately disturbed function, "Function C": highly disturbed function.

Figure 5 demonstrates the annual change in liver function of hepatectomized patients. The number of hepatectomies is shown by three groups of liver function; "function A" (almost normal), "function B" (moderately disturbed) and "function C" (highly disturbed). In Figure 5, there is an increase in cases with "function B". The total numbers of hepatectomized cases in these functional groups were 141 (33.0%, 226 (52.9%) and 60 (14.1%), for "function A", "B" and "C", respectively.

*Surgical Procedure*

The surgical procedures adopted in this series classified as non-anatomical resection, sub-segmentectomy, mono-segmentectomy, bi-segmentectomy and tri-segmentectomy. The hepatic "segments" are those defined by Healey[2] as the right posterior, the right anterior, the left medial and the left lateral segment. "Sub-segmentectomy" is defined as an anatomical resection smaller than one hepatic segment[3]. "Non-anatomical" resection is, in general, a small resection. However, the difference between "non-anatomical' and "sub-segmentectomy" is whether or not it is anatomical. The numbers of these surgical procedures are shown in Table 2. The annual change in the surgical procedures are shown in Figure 6. The increase of the number of small range resection such as non-anatomical resection and subsegmentectomy is remarkable, although the number of mono-segmentectomy and bi-segmentectomy increased a little with a rise with the total number of resections.

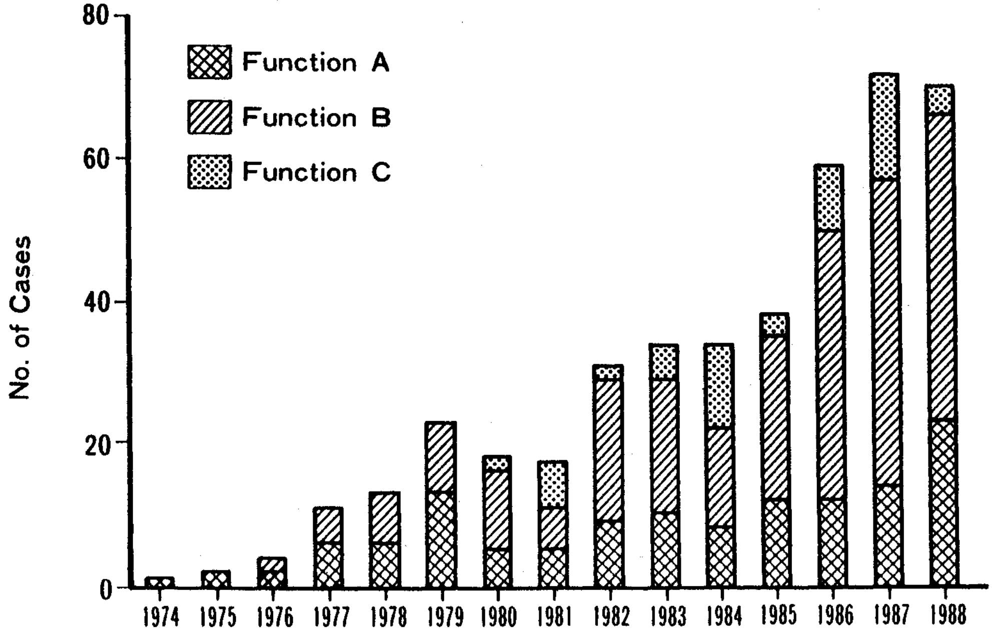

**Figure 5** The annual trend of liver function in the hepatectomy cases. The definition of liver function, A, B, and C, are shown in the Figure 4.

**Table 2** Surgical procedures for HCCS

| *Surgical Procedures* | *No. of Cases (%)* |
|---|---|
| Non-anatomical resection | 197 (46.1%) |
| Sub-segmentectomy | 101 (23.7%) |
| Mono-segmentectomy | 44 (10.3%) |
| right posterior | 21 |
| right anterior | 7 |
| left medial | 1 |
| left lateral | 15 |
| Bi-segmentectomy | 62 (14.5%) |
| right | 31 |
| left | 17 |
| central | 14 |
| Tri-segmentectomy | 23 (5.4%) |
| right | 19 |
| left | 4 |
| Total | 427 |

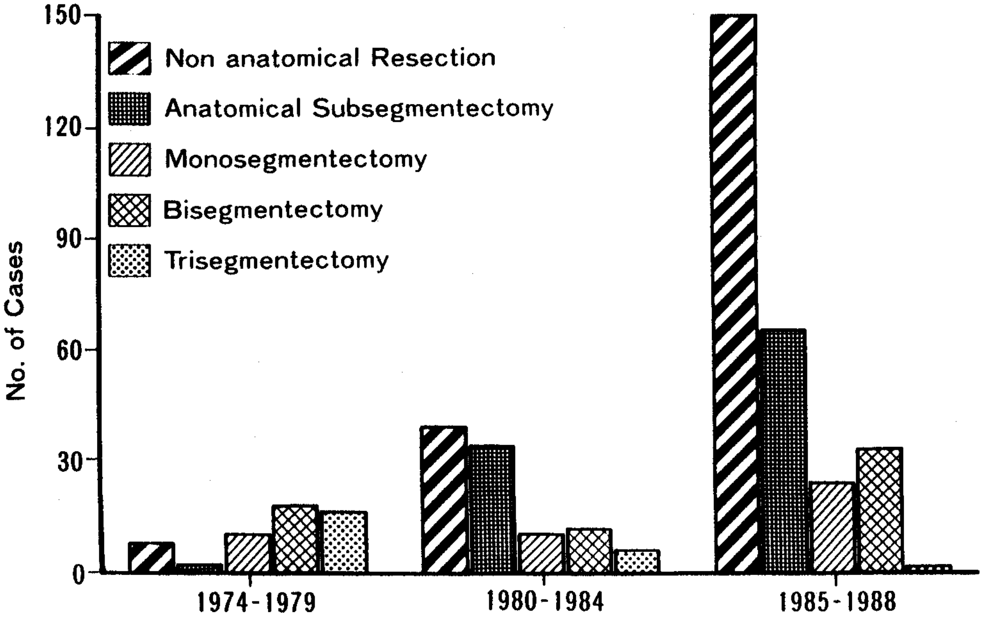

**Figure 6** The numbers of surgical procedures in each period.

*Operative Mortality Rate*

The overall mortality rate was 3.3% (14/427) between 1974 and 1988 (Figure 7). The period was divided into two halves, the earlier half, 1974–1981, and the latter, 1982–1988. The mortality rate in the earlier stage was 10.1% (9/89) and that in the latter was 1.5% (5/338). This difference was significant ($p<0.01$).

*Survival Rate After Hepatectomy*

The postoperative survival rate was calculated at the end of 1988 by the Kaplana-Mier method. The cases of operative mortality (deaths within 30 post-operative days) were included. However, cases of non-curative resection and cases of re-resection were excluded. There were 50 survivors longer than 5 years and 6 out of 50 are presently surviving more than 10 years, (May, 1989).

*Overall cases*

The overall survival rates (mean % ± SD) in 378 patients including operative mortalities and excluding palliative resections and re-resections, were 82.9% ±2.0, 69.5% ±2.6, 57.0% ±3.1, 45.0% ±3.5, 40.6% ±3.7, 28.9% ±4.4 and 22.7% ±5.3 for 1, 2, 3, 4, 5, 7, and 10 year, respectively (Figure 8).

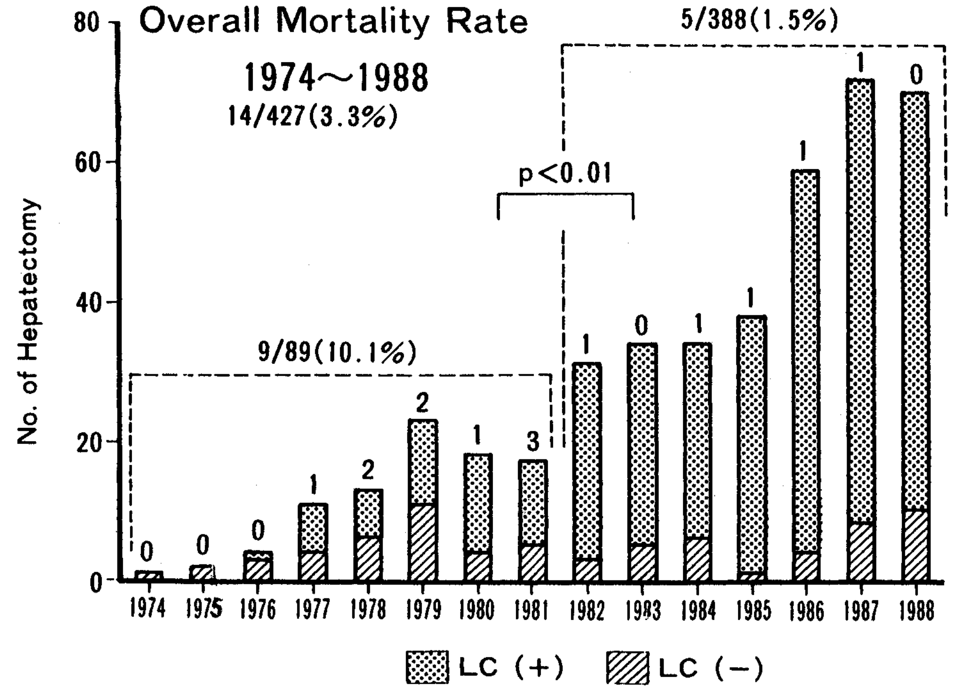

**Figure 7** The operative rates (within 30 postoperative days) in hepatectomy for HCC. The differences of the mortality rate between 1974–1981 and 1982–1988 are significantly ($p<0.01$). The number above each column is the number of death(s) in each year.

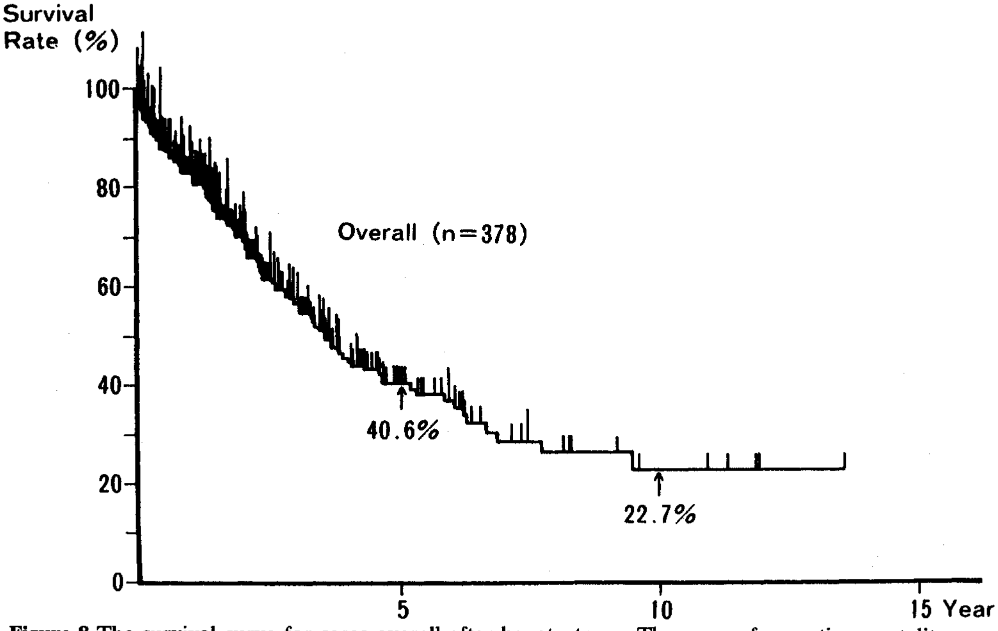

**Figure 8** The survival curve for cases overall after hepatectomy. The cases of operative mortality are included and those of non-curative resections and reresections are excluded. The definition of "non-curative resection" is described in the text.

*Survival rate by cirrhotic or non-cirrhotic*

The survival rates of the cirrhotic patients(n = 315) and the non-cirrhotic (n = 63) were calculated separately (Figure 9). Although the shapes of the survival curves of these two groups are different, the 5 year survival rates were the same.

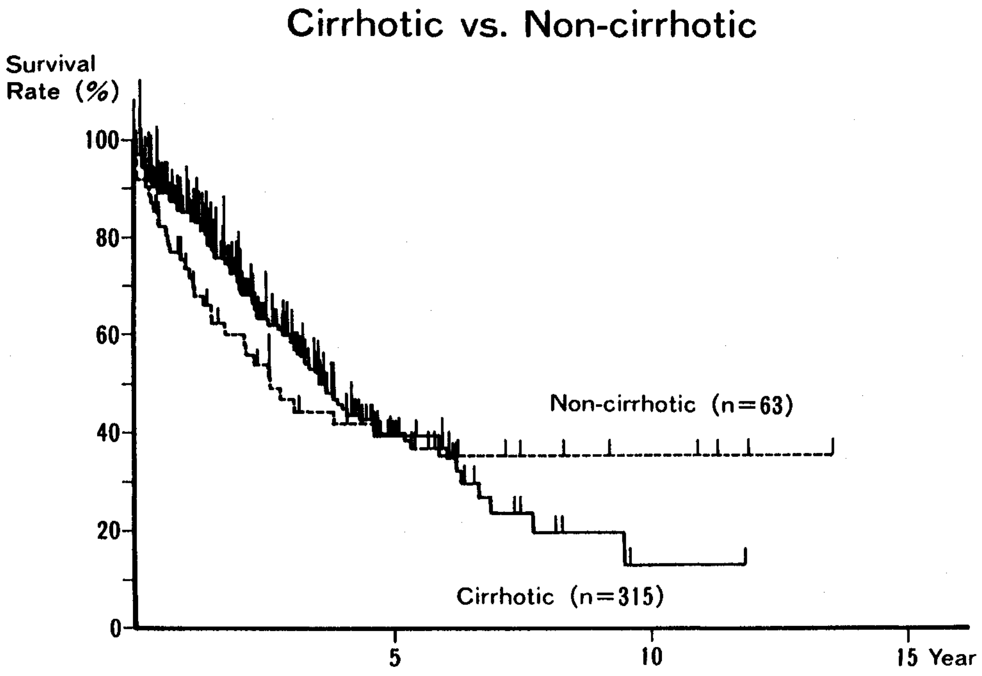

**Figure 9** A comparison of survival rate between the non-cirrhotic and cirrhotic patients. During 1–432 days, the survival rate of the cirrhotic group was good however during 3888–4320 days the survival rate of the non-cirrhotic group was better ($p < 0.05$).

*Difference in survival by year of operation*

The difference in the survival rate between the patients operated on before 1981 (n = 78) and after 1982 (n = 300) has been discussed (Figure 10). Three, 5, and 10 year survival rates of the patients operated on before 1981 were 37.2% ± 5.5, 25.6% ± 4.9 and 16.1% ± 4.6, respectively. Those of the patients after 1982 were 63.2% ± 3.6 for 3 years and 46.1% ± 4.8 for 5 years. The difference was significant at 1–5 years($p < 0.05$).

### DISCUSSION

The number of hepatectomies in recent years has been increasing greatly. Surgery for HCC in the normal liver have not increased. However, the increase in hepatectomy rate for HCC associated with liver cirrhosis is remarkable. The

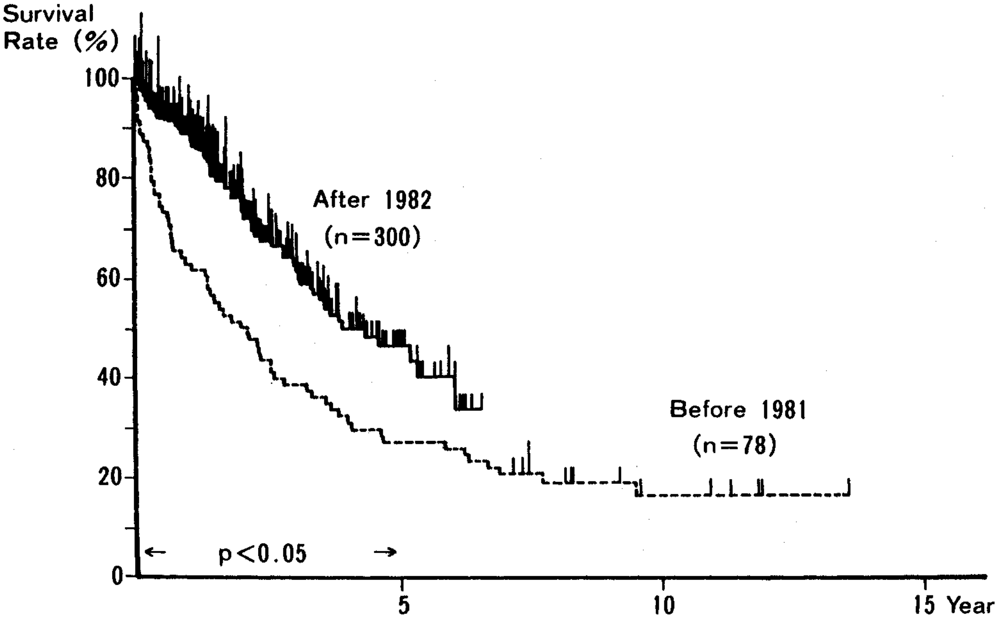

**Figure 10** The differences in the survival rate between the patients operated on before 1981 and that after 1982. During 1–1904 days, the difference was significant (1–952) days: $p<0.001$, 953–1666 days: $p<0.01$, 1667–1904 days: $p<0.05$).

concept, that chronic liver disease, such as chronic hepatitis or cirrhosis is an important aspect in the management of HCC, is well established throughout Japan. Fortunately, at the same time, modern technology provided some powerful tools which can rationalize the concept. Around 1980, US and CT were introduced into clinical medicine. Surgeons were made aware of an increase in the number of operable cases with HCCs, especially HCCs of small size (Figure 1). Since 1982, the number of hepatectomies for HCC with LC has been increasing. The increase in numbers of small HCC less than 5cm in diameter after 1982 is outstanding (Figure 2). And at the same time, these modern diagnostic techniques picked up the second or third foci in the same liver. The incidence of cases with small multiple nodules was the same as those with larger HCCs (Figure 3).

Small HCCs are detected during screening for chronic liver disease. Almost all small HCCs are detected in the injured liver. It is now routine to tackle hepatectomy in patients with disturbed liver function. As summarized in Figure 4, more than 80% of our hepatectomy cases were cirrhotic and about 80% of these cirrhotic cases had HCCs 5cm or less in diameter. Thus, 2/3 of our cases were small HCCs in cirrhosis. However, with the advancement in surgical technique, mainly saving in blood loss (Figure 11), the indications for hepatectomy in the injured liver have been expanded. The number of operations in the injured liver are increasing yearly as depicted in Figure 5. The increase in small HCCs in the injured liver is associated with a change in the surgical procedures carried out. During 1970s, more than half of our hepatectomies were extended resections such as bi- or tri-segmentectomy (Figure 6), in recent years the numbers of non-anatomical resection and subsegmentectomy have increased.

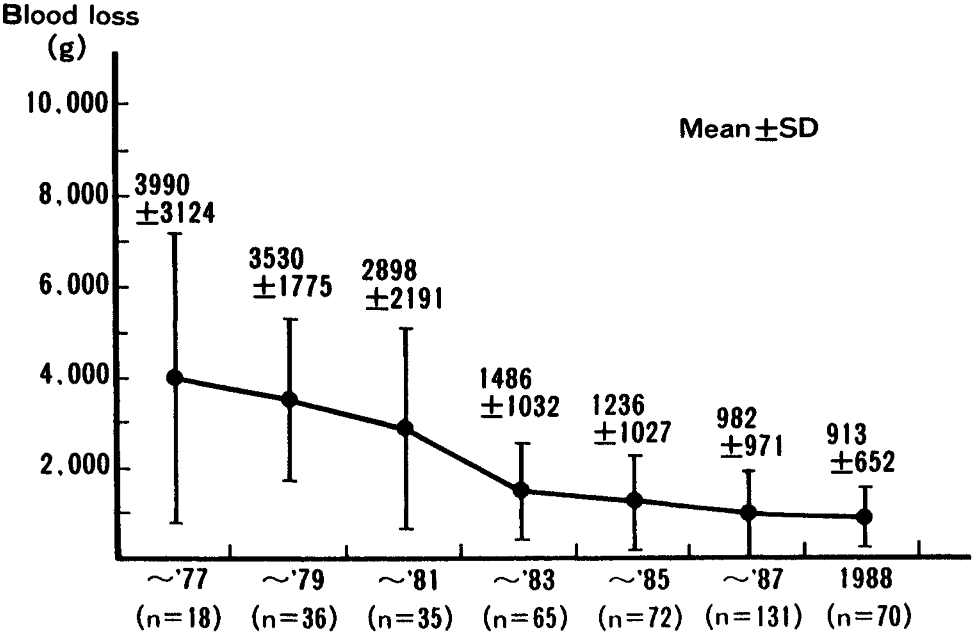

**Figure 11** The volume of blood loss in hepatectomy. The difference of the mean value from 1974 to 1981 was significantly more than the latter years ($p < 0.05$).

The operative mortality rate has improved in the latter half of this series, in spite of the increase of cases with poor liver function. This also corresponds to the decrease of the mean value of operative blood loss per annum (Figure 11). We adopted the vascular clamping technique around 1981. At the time of parenchymal transection, the hepatic artery and the portal vein at the porta hepatis were clamped. This procedure effectively decreases blood loss. In the cirrhotic patients, the mean value of blood loss among who survived surgery ($n = 345$) was 1394.7 ± 159.7ml (mean ± SD). On the other hand, those who died within 30 postoperative days ($n = 9$), had a blood loss of 3529.4ml ± 1954.2. This difference was significant($p < 0.01$, two tailed t-test).

The survival rates after hepatectomy overall were 40.6% for 5 year and 22.7% for 10 year at the end of 1988.

The influence of combined chronic disease on survival was examined. Some surgeons have reported the results of hepatectomy comparing cirrhotic and non-cirrhotic patients. However, the conclusion is still controversial[4–8]. The survival rates were calculated in the non-cirrhotic group ($n = 63$) and in the cirrhotic group ($n = 315$) separately (Figure 9). The 5 year survival rates were the same (39.3% vs. 39.9%) between the two groups. However, the shapes of the survival curves are somewhat different. The curve of the non-cirrhotic group levelled out around 4 or 5 years and there was no death after 5 years. This is a common pattern for the survival curve in many other visceral cancers. This may suggest that the prognosis of the non-cirrhotic patients depends solely upon the status of the primary cancer, on the contrary, the curve of the cirrhotic patients continues to go down to around

10 years. This is peculiar to the disease, HCC with cirrhosis. Thus, it may suggest that the destiny of the patients depend not only upon the status of the primary cancer but also upon other factors, such as liver function and multiple genesis of HCC in cirrhosis.

The survivals were compared between the earlier half (1974–1981) of this series and the latter half (1982–1988). As shown in figure 10, the result of the latter half (n = 300) was clearly better. Before concluding that "these data suggest the treatment of HCC has improved recently", the background of the patients of each period should also be compared. The cases were divided by the TNM stage[9] (Figure 12). In the group of patients operated on before 1981, there was no case of stage 1 and the numbers in other three stages, stage 2, 3 and 4, were almost even. In the latter group, operated on after 1982, the number of cases in stage 3 has increased. However, the proportion of stage 1 and 2 after 1982 has increased significantly.

There were more cases of an early stage of cancer in the latter half, but from the point of view of liver function (Figure 13), there were more cases with injured liver function in the latter half. The author feels that the survival rates should be compared between cases in a same stage of the cancer and with the same liver function. Figure 14 shows a comparison of the survival rates between the cases with HCC 5cm or less in diameter and in function B, operated on before 1981 and after 1982. The differences between the two survival curves are significant by the generalized Wilcoxon test ($p < 0.001$). Here, it can be said that the recent improvement of the survival rate in the patients is due not only to "early detection" but also to "progress of treatment".

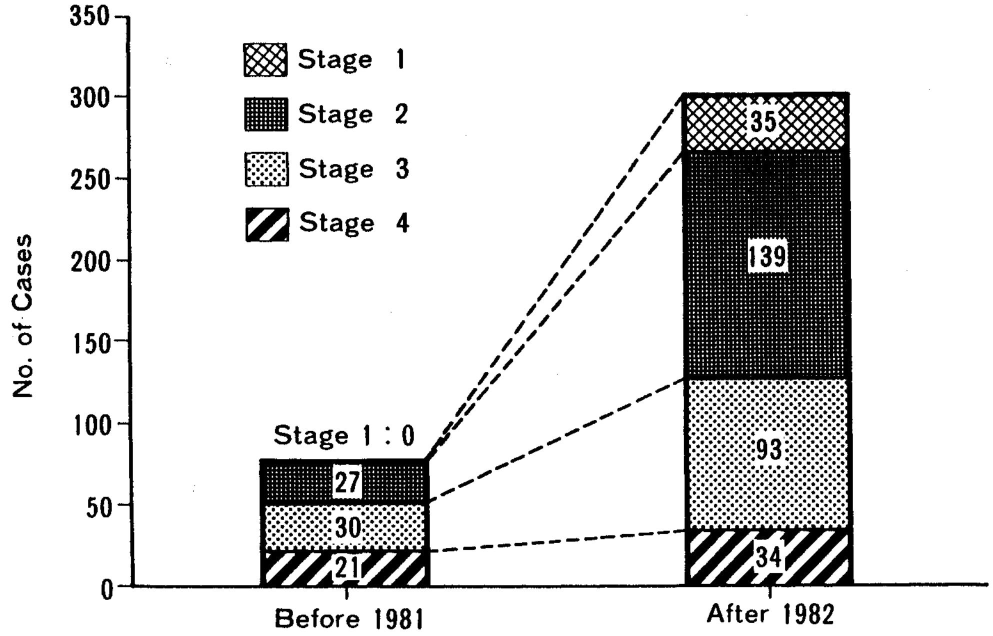

**Figure 12** The number of cases by the TNM classification of liver cancer (UICC) in the earlier and latter period of this series. There was no case of Stage 1 in the earlier period.

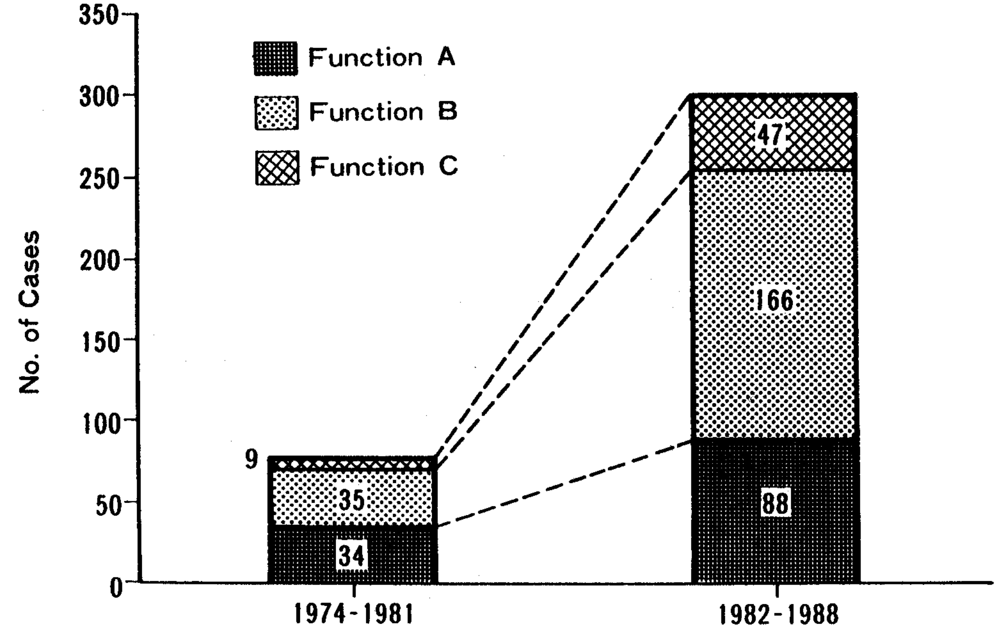

**Figure 13** The change in the number of cases by liver function as defined in igure 4. During 1974–1981 the numbers of "Function A", "B" and "C" were 34, 35 and 9 and during 1982–1988 88, 166 and 47, respectively.

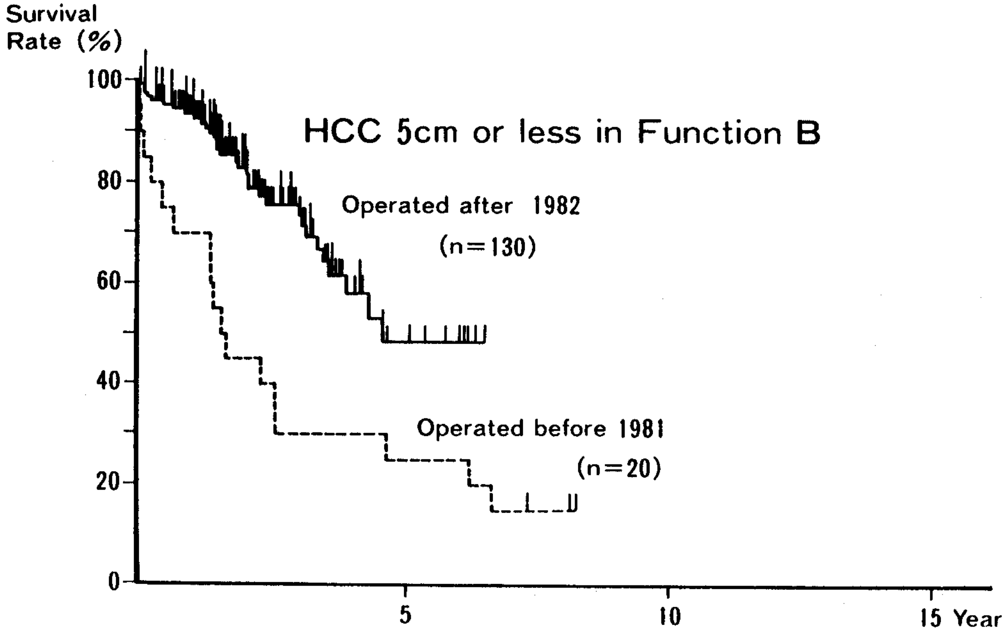

**Figure 14** The survival rates of patients with HCC 5cm or less in "Function B". The difference between the group operated on before 1981 and that after 1982 was significant by the generalized Wilcoxon test ($p<0.001$).

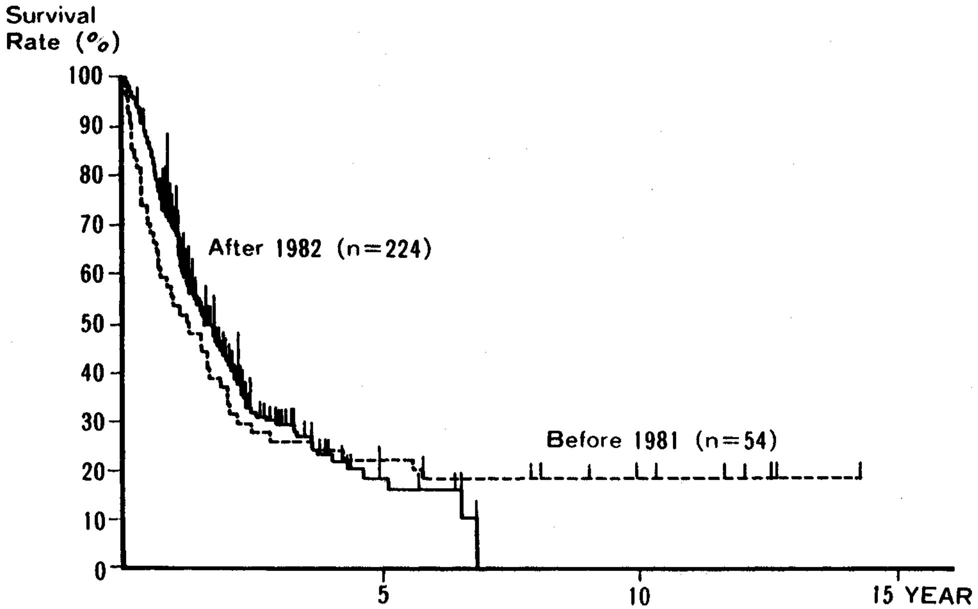

**Figure 15** The cancer-free survival rates after hepatectomy of the patients operated on before 1981 and after 1982. The cases of operative mortality, hospital death, death from non-HCC, active double cancers in other organs and insufficient follow-up were excluded. There was no significant difference.

To verify "progress of treatment", some other analyses were added. In Figure 15, the cancer-free survival rates of the earlier and the recent group are shown. The cancer-free survival rates of these two groups were almost the same, although in the recent group, there were more cases with HCCs of the earlier stages as shown in Figure 12. This may suggest that the small range resections such as non-anatomical resection and subsegmentectomy for the small HCCs in the injured liver which were carried out in the recent group were not sufficiently radical. At the same time, this may suggest a higher than expected incidence of multicentric carcinigenesis of HCC in the injured liver. In Figure 16, the survival after the detection of recurrence is compared between the two groups, the early and the recent stage. In this analysis of the survival rates, the cases of operative mortalities, hospital deaths, deaths of non-HCC, active double cancers of other organs and insufficient follow-up were excluded. Howeve although the cancer-free survival rates were the same, the survival rates were different, which means in the recent group the cancer-bearing survival term has been prolonged. This shows the treatment for recurrences in recent years is more effective than in the earlier stage. In the earlier stage of this series, re-resection was the only effective treatment for recurrence, but recently not only re-resection but arterial embolization and ethanol injection therapy have been adopted. The possibility that, in the recent group, early detection of a recurrent lesion may be associated with an apparent longer survival may not be neglected. The prolongation of the survival in the recent cases must be a fruit of these multiple factors.

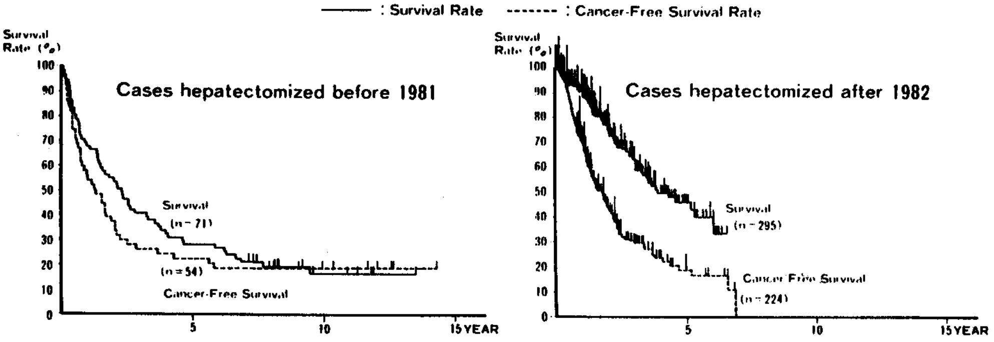

**Figure 16** A comparison of the survival rate and the cancer-free survival rate in the cases operated on before 1981 (left) and in those after 1982 (right). The subjects of these analyses were the same as those in the Figure 15.

### *References*

1. Child, C.G. (1964) The liver and portal hypertension, p.50, Philadelphia, MPCS
2. Healey, J.E.Jr. and Schroy, P.C. (1953) Anatomy of the biliary duct within the human liver, Analysis of the prevailing pattern of branching and the major variations of biliary ducts. *Archives of Surgery*, **66**, 599–616
3. Yamasaki, S., Makuuchi, M. and Hasegawa, H. (1981) Clinico-pathological observation of the minute liver cancer and the new method of hepatectomy; Analysis of 27 resected cases. *Acta Hepatologica Japonica* **22**, 1714–1724, (in Japanese, English abstract)
4. Zhou, X.D. and Tang, Z.Y. (1985) Factors influencing resectability and resection survival rates of subclinical hepatocellular carcinoma, in Subclinical hepato-cellular Carcinoma, edited by Tang, Z.Y., pp. 78–84, Beijing. China Academic Publishers
5. Nagasue, N., Yukaya, H., Ogawa, Y., Sasaki, Y., Chang, Y.C. and Miimi, K. (1986) Clinical experience with 118 hepatic resections for hepatocellular carcinoma, *Surgery*, **99**, 694–702
6. Kim, S.T. (1988) Clinical analyses of 127 patients who underwent hepatic resection for primary hepatocellular carcinoma. The Netherlands Journal of Surgery, Abstracts of 2nd World Congress on Hepato-Pancreato Biliary Surgery, FP129
7. Saito, Y., Ohyanagi, H., Usami, M., Kanamaru, T., Hanabata, M. and Gu, E. (1987) The long term survivals of various therapies on the primary liver cancer. *Gastroenterological Surgery*, **10**, 797–802, (in Japanese)
8. Yamasaki, S., Makuuchi, M. and Hasegawa, H. (1988) Present status in Cancer Therapy, Liver Cancer. *KARCINOS*, **1**, 41–52, (in Japanese)
9. International Union Against Cancer (1987) Liver in TNM Classification of Malignant Tumours, 4th edn, edited by P. Hermanek and L.H. Sobin, p.53–55, Geneva: Springer-Verlag

*(Accepted by S. Bengmark 1 July 1990)*

